# Peroxisomes and the nervous system: progress and challenges in neuroinflammation and cell-specific functions

**DOI:** 10.3389/fnmol.2026.1745086

**Published:** 2026-02-11

**Authors:** Francesca Di Cara, Stéphane Savary

**Affiliations:** 1 Department of Microbiology and Immunology, Dalhousie University, Halifax, NS, Canada; 2 Université Bourgogne Europe, Institut Agro, CNRS, INRAE, UMR CSGA, Equipe NeuroFeed, Dijon, France

**Keywords:** lipid metabolism, microglia, nervous system, neuroinflammation, peroxisome signalling

## 1 Introduction

The role of peroxisomes in brain development has been evident for many decades following the discovery that defects in peroxisome biogenesis lead to Zellweger Spectrum Diseases (ZSD) which presents neurodevelopmental failure (Faust et al., 2005; Berger et al., 2016). Peroxisomes perform essential biochemical functions, including the synthesis of omega-3 polyunsaturated fatty acids (PUFAs), which are critical for brain and neuronal health, and the catabolism of very long-chain fatty acids (VLCFAs) and reactive oxygen and nitrogen species. These processes are particularly important in the brain, where metabolic imbalances or toxic accumulations can disrupt development and function (Carmichael, 2025). Growing evidence demonstrates that peroxisomal metabolites and signaling functions are required in various brain cell types, including neurons, microglia, and oligodendrocytes. This suggests that peroxisomes support brain health through both cell-type-specific and broader metabolic mechanisms. Moreover, extra brain peroxisomal activities, for instance in the liver, are also critical for brain development and function, and their impairment can contribute to diseases such as ZSD or intellectual disabilities (Janssen et al., 2003). A recent work also suggests that peroxisomes may regulate the microbiota-gut-brain axis. Indeed, a supplementation with *Lactobacillus acidophilus* improved outcomes in a mouse model of cerebral ischemia by increasing intestinal absorption of linoleic acid that reaches the brain and activates peroxisomes in microglia, promoting their shift to an anti-inflammatory state (Huang et al., 2025). This work also opens the possibility that peroxisomes in the intestinal epithelium may contribute to the production of metabolites that control brain functions or inflammatory status. This concept may extend to crosstalk between the brain and other peripheral tissues, such as adipose tissue or muscle.

Moreover, research in neurodegenerative diseases has also found that peroxisome metabolites are required to maintain a healthy brain over aging, and alterations in the brain or in the circulation of peroxisomal lipids have been linked to the onset of neurodegenerative conditions such as Alzheimer's and Parkinson's disease (Fabelo et al., 2011; Lizard et al., 2012; Roczkowsky et al., 2025). This is in line with the recent demonstration that impaired peroxisomal β-oxidation contributes to microglial dysfunction, linking peroxisomes to the progression of Alzheimer's disease (Gao et al., 2025). Yet, whether the mechanisms of health/disease of the brain during aging are neuronal-specific, brain-specific, or of extra-brain origins remains understudied. Here, we comment on current knowledge linking peroxisomes to brain health, suggesting new directions and technologies to expand this crucial and interesting area of the role of peroxisomes in brain physiology.

## 2 Cell-specificity of peroxisomes and peroxisomal metabolism in the brain

Peroxisomes exhibit remarkable abundance and functional diversity across the various cell types of the nervous system, encompassing neurons, astrocytes, oligodendrocytes, and microglia (Deb et al., 2021). Deciphering the precise functional roles of peroxisomes within individual cell types remains a significant scientific challenge. While peroxisomes are known to play an essential role in lipid metabolism, redox balance, and cell signaling, their specific activities and the consequences of their dysfunction can vary considerably depending on the tissue and cellular context. To address this complexity, tissue-specific and cell-type-specific knockout models have become invaluable tools (Baes et al., 2002; Kassmann et al., 2007; Krysko et al., 2007; Baes and Van Veldhoven, 2012; Verheijden et al., 2014; Di Cara et al., 2017; Beckers et al., 2018, 2019; Raas et al., 2019a,b, 2023; Tawbeh et al., 2023, 2025). While these approaches have provided critical insights into the role of peroxisomes in neurons, astrocytes, oligodendrocytes, and microglia, many questions remain unanswered.

The expression pattern of peroxisome proliferator-activated receptors (PPARs), nuclear receptors involved in peroxisome proliferation and controlling the expression of many peroxisomal genes, likely reflects the importance of peroxisomes and their ability to adapt their number and the nature of their protein content to various stimuli (Kainu et al., 1994; Braissant et al., 1996; Woods et al., 2003). Notably, PPAR-dependent regulation of peroxisomes is highly species-specific, with marked differences between rodents and humans, which has important implications for translating findings from animal models to human brain physiology (Mast et al., 2025). In the brain and despite their ubiquity, the cell-type-specific functions of peroxisomes and their dynamic adaptations depending on aging, nutritional environment, or pathological stimuli remain elusive. Many studies based on *in situ* hybridization and immunohistochemistry have significantly contributed to highlight the key roles of peroxisomes in the brain; however, these studies were usually limited to a single specific target or a specific pathological condition and did not provide a dynamic view or an understanding of the differences existing between peroxisomes depending on the cells, organ areas, and contexts. Among these, there are studies focusing on the expression of ABCD1-3 proteins, which transport various lipidic substrates through the peroxisomal membrane (Pollard et al., 1995; Troffer-Charlier et al., 1998; Fanelli et al., 2013), NOS2, the inducible nitric oxide synthase partially localized in the peroxisome (Gilg et al., 2000), PEX14, a translocon protein and Catalase, the enzyme in charge of hydrogen peroxide catabolism (Semikasev et al., 2023) in distinct areas. The upcoming implementation of new cutting-edge single-cell RNA sequencing and spatial transcriptomics technologies, such as RNAscope, holds significant promise for precisely elucidating the cellular distribution of peroxisomal gene expression (Piwecka et al., 2023; Secci et al., 2023). Spatial mass spectrometry [matrix-assisted laser desorption/ionization (MALDI) or desorption electrospray ionization (DESI)] is another powerful technique to visualize the spatial distribution and dynamics of peroxisomal proteins in cellular contexts, thereby helping in elucidating the cellular and physiological role of peroxisomes in the nervous tissues (Gessel et al., 2014), especially when combined with other high-throughput special analyses such as spatial lipidomics (Opielka et al., 2025). The combination of these approaches would also inform us about the relationships of peroxisomes with other cellular compartments, helping to define the poorly understood metabolic fluxes in which they participate. Peroxisomes physically interact with ER, mitochondria, lysosomes, and lipid droplets, and are now emerging as signaling hub organelles coordinating lipid metabolism, redox homeostasis, thermogenesis and inflammatory responses (Park et al., 2019; Di Cara et al., 2023; Kumar et al., 2024). Understanding the dynamics of lipid turnover for metabolic reprogramming of organelles and the synthesis or the degradation of signaling lipids such as eicosanoids will be critical to anticipate and describe neuronal and glial functions in health and disease status.

## 3 Peroxisomes at the crossroads of brain neuroinflammation: linking microglia and disease

Regarding inflammatory response in nervous tissues, that can be both beneficial and detrimental depending on the context and the timing of the response, the role of peroxisomes has often been underrated. Several studies have highlighted the importance of peroxisomes in producing and releasing pro-inflammatory cytokines such as TNF or IL1B (Nath et al., 2022; Roczkowsky et al., 2025). However, a comprehensive understanding of their role throughout the different phases of the inflammatory response, from initiation to resolution, remains lacking. This is also true for the dynamics of inflammatory mediators. Peroxisomal lipid metabolism also contributes to the production of lipid mediators such as PUFAs, eicosanoids, and docosanoids (Savary et al., 2012). However, the cellular cascades regulating the production of these lipids in peroxisomes, and the mechanisms governing their dynamics remain undefined.

Microglia, through their plasticity, their diversity of function, and their ability to migrate and communicate with other brain cells, are obviously central in the brain homeostasis (Muzio et al., 2021). Regarding the pathogenesis of peroxisomal disorders, especially X-linked adrenoleuokodystrophy, reconciliating biochemical observations such as VLCFA accumulation, cell toxicity, inflammation, demyelination, and neurodegeneration, and providing a comprehensive sequence of events explaining the disease evolution in patients, constitutes a true challenge. Recent findings have highlighted the key role of peroxisomes in microglia and their involvement in various cellular processes such as phagocytosis of myelin and cell signaling (Raas et al., 2023; Tawbeh et al., 2023; Barnes-Velez et al., 2025; Tawbeh et al., 2025).

These insights have generated new hypotheses regarding our understanding of the pathogenesis of peroxisomal diseases, which place microglia and their communication with neurons as a key alteration in disease onset (Bergner et al., 2019; Yska et al., 2024). Of course, it does not exclude a role for other glial cell types or extracellular vesicles (EVs). EVs could participate in long-range intercellular communication by transporting reactive oxygen species, lipids or other signaling molecules generated in response to peroxisomal alterations, and possibly entire organelles (Ghadami and Dellinger, 2023). This could facilitate the propagation of dysfunctional signals, potentially exacerbating disease progression. Conversely, EVs, and not only the supposed functional replacement of microglia by healthy macrophages, could be a key component explaining how hematopoietic stem cell gene therapy contributes to halting disease progression (Mallack et al., 2019), a hypothesis that warrants further study.

## 4 Discussion

Recent research has brought to light the complexity in understanding the requirement for peroxisomes and their metabolism for brain health. It is obvious that regionality, cell-specificity, and local vs. distal signaling need to be analyzed and considered to define the peroxisomal role in brain health and, most importantly, to identify the alteration of peroxisomal signaling that impacts brain development and functions in various pathological conditions. Our commentary hopes to direct research in the field toward more integrated analyses and exploring multiple model organisms to advance the field and address outstanding questions such as:

- What are the cell-specific peroxisomal contributions in the developing and adult brain?
- How do peroxisomes outside the brain impact developmental neurogenesis and brain health?
- Are peroxisomes fundamental players in the microbiota-gut-brain axis?
